# Prevalence and risk factors of chlamydia infection in Hong Kong: A population-based geospatial household survey and testing

**DOI:** 10.1371/journal.pone.0172561

**Published:** 2017-02-22

**Authors:** William Chi Wai Wong, Yanping Zhao, Ngai Sze Wong, William L. Parish, Heidi Yin Hai Miu, Li-Gang Yang, Michael Emch, King Man Ho, Francois Yeung Fong, Joseph D. Tucker

**Affiliations:** 1 Department of Family Medicine and Primary Care, Li Ka Shing Faculty of Medicine, The University of Hong Kong, Hong Kong S.A.R., China; 2 University of North Carolina Project-China, Guangzhou, Guangdong Province, China; 3 Institute for Global Health & Infectious Diseases, University of North Carolina at Chapel Hill, Chapel Hill, United States of America; 4 Sociology Department, University of Chicago, Chicago, Illinois, United States of America; 5 Guangdong Provincial Centers for Skin Diseases and STI Control, Guangzhou, Guangdong Province, China; 6 Department of Geography, University of North Carolina-Chapel Hill, Chapel Hill, North Carolina, United States of America; 7 Social Hygiene Service, Department of Health, Hong Kong Government, Hong Kong S.A.R., China; 8 Hong Kong Sexual Health Centre, Hong Kong S.A.R., China; 9 London School of Hygiene and Tropical Medicine, London, United Kingdom; Albert-Ludwigs-Universitat Freiburg, GERMANY

## Abstract

**Background:**

Chlamydia causes infertility and increases risk of HIV infection, and population-based studies provide essential information for effective infection control and prevention. This study examined *Chlamydia trachomatis* prevalence and risk factors among a representative sample of 18-49-year-old residents in Hong Kong.

**Methods:**

Census boundary map of 412 constituency areas was used as primary sampling units to construct the sampling frame and, residential buildings and units were randomly selected using geospatial modelling. A questionnaire on sexual practice and health was conducted, and polymerase chain reaction was used to test the urine for genital chlamydial infection. Invitation letters were sent to the selected households and a team of interviewers were sent to recruit one subject per household. Prevalence data was weighted according to the 2011 census and risk factors identified through logistic regression.

**Results:**

Among 881 participants (response rate of 24.5%), the overall *Chlamydia trachomatis* prevalence was low at 1.4% (95%CI 0.8–2.5%) but sexually active young (18–26 years) women had relatively high prevalence (5.8%, 95%CI 1.7–18.2%) in Hong Kong. A unique U-shape disease burden was observed with peaks in younger and older (40–49 years) women. Amongst the sexually active women, the risk factors of *Chlamydia trachomatis* infection were: younger age (aOR = 25.4, 95% CI 2.81–230); living alone (aOR = 8.99, 95% CI 1.46–55.40); and, among all the sexually active participants, males (including the male partners of the female participants) who had travelled out of Hong Kong in the previous 12 months had higher risks of infection (aOR = 5.35; 95% CI 1.25–22.8). A core-peripheral geographical distribution of *Chlamydia trachomatis* prevalence was also observed.

**Conclusion:**

Young and older sexually active women in Hong Kong have high prevalence of chlamydia. Routine screening for sexually active women and young men should be considered. Further research on testing feasibility and linkage-to-care are urgently needed to control the infection.

## Introduction

Chlamydia is the most common notifiable disease in the European Union [1] and USA [2]. In 2014, over 1.4 million cases of chlamydia were reported to US Center for Disease Control and Prevention (CDC), the highest number of any infections ever reported [2]. It was estimated in 2008 that the lifetime direct medical cost of chlamydia had reached US$516.7 million (excluding indirect costs such as loss of productivity and other intangible costs) [3]. Up to 80% of women with genital chlamydia is asymptomatic, but itself can be both highly contagious and dangerous in terms of long-term health consequences such as pelvic inflammatory disease, infertility and ectopic pregnancy [4, 5] as well as increased risks of HIV infection [6]. All these complications can be avoided with early diagnosis and treatment.

Representative population-based prevalence data are the crucial first step in understanding the disease burden and its spread, creating the foundation for effective intervention, prevention and other control measures such as resource allocation, designing and delivery [7, 8]. These studies have been undertaken extensively in Europe [8–13], USA [14, 15], and China [16]. The National Surveys of Sexual Attitudes and Lifestyles (Natsal) from the UK has provided useful epidemiological data to inform guidelines in England [8], Scotland [17], and Wales [18]. Likewise, the US Preventive Services Task Force (USPSTF) has also recommended screening of all sexually active females below 25 years old since 2007 [19], again reinforced in 2014 [20].

With a population of 7.3 million people, Hong Kong is an important global city with frequent exchange with other parts of the world [21]. The epidemiological significance of this was evident in the Severe Acute Respiratory Syndrome (SARS) and avian flu outbreaks in recent history. It was estimated from a recent review and meta-analysis that the prevalence of travel-associated casual sex could be as high as 20%, about half were unsafe sex without condoms [22]. Amongst the 60 million annual visitors to Hong Kong, 77% of them were from mainland China [23]. Concurrently, sexually transmitted infections (STIs) have risen rapidly in mainland China; for examples, syphilis incidence has nearly tripled from 12.80 per 100,000 persons in 2006 to 31.85 per 100,000 in 2015 [24, 25]. Incidence caused by genital *Chlamydia trachomatis* (CT) has increased by nearly 40% from 37.20 per 100,000 persons in 2010 and 51.3 per 100,000 in 2014 in the neighbouring Guangdong Province [26, 27].

Hong Kong has never been included in any population-based STI prevalence studies and STIs are not notifiable locally. As a result, the limited data on STIs are either from key populations (e.g. female sex workers)[28] or specific settings such as government-run Social Hygiene Clinics (SHCs). Anecdotally an increasing trend of STIs was observed; for example, from 2011 to 2015, the STI cases diagnosed at SHCs had increased by 9.8% whilst primary and secondary cases of syphilis had been more than doubled [29, 30]. This study examined the prevalence of *Chlamydia trachomatis* infectionand risk factors among a population-based sample of 18-49-year-old residents in Hong Kong.

## Methods

### Sampling design

Territory-wide STI and Sexual Health Survey (TeSSHS) was a population-based, geospatial representative household survey characterised by computer-assisted personal interview (CAPI) questionnaire and, *Chlamydia trachomatis* and *Neisseria gonorrhea* polymerase chain reaction (PCR) testing between November 2014 and March 2016. Hong Kong consists of 18 districts subdivided into 412 District Council Constituency Areas (DCCAs) to contain approximately 17,000 residents in each DCCA [31]. Hence, participants were drawn from randomly selected 79 primary sampling units based on theseDCCAs in proportion to the total number of DCCAs within each district.

Using Geospatial Modelling Environment software, random points were dropped in accordance to the 2011 Hong Kong census boundary maps. These points were then matched to proximally-located residential buildings, and a proportional number of households (roughly between 7–20 households) relative to the buildings were randomly selected. Only one eligible subject per household was permitted to participate in the study in order to reduce intra-class correlation of family members from the same household. The sample size for TeSSHS was based on the prevalence of 2.3% identified in the Chinese Health and Family Life Survey [16] and therefore, to detect an standard error of 0.01, a sample size of 863 participants were estimated. The Institutional Review Board (IRB) of the University of Hong Kong/Research Ethics Committee of the Kowloon West Cluster, Hospital Authority of Hong Kong approved this project with the name “To Determine Prevalence and Contextual Risk Factors of Sexually Transmitted Infections in Hong Kong” (IRB Reference Number UW13-058).

### The questionnaire

The survey instrument was based on the Chinese Health and Family Life Survey, which was a validated questionnaire used in the previous *Chlamydia trachomatis* prevalence study in China [16]. The questionnaire was then matched and appraised according to a review of reviews of STI/HIV risk factors among the Chinese population [32]. It covered areas regarding demographic information, health and health behaviours, sexual practice and sexual health as well as partner’s travel history for female participants. The questionnaire was pilot-tested twice and modified before its launch.

The survey was conducted mostly at the participants’ home or through appointments in one of the four centres across the city. Initially interviewer-assisted, a notification indicates when the survey switches to the self-completed components for the participants to complete confidentially. After that, the participants were asked to provide the first part of the urine stream in a given sample container for PCR testing. Only those who had completed the questionnaire and provided the urine samples were considered successful recruits.

### PCR testing for *Chlamydia trachomatis*

All the test samples were taken to the laboratory by the interviewers within a week to be tested for *Chlamydia trachomatis* and maintained at 4°C if any storage and transportation were necessary. Cool packs were used for transportation during the summer months in Hong Kong. 15 ml of urine sample was centrifuged at 1500g for 10 minutes to collect epithelial cells. Cell pellet was washed with 10 ml of PBS to remove any debris. Nucleic acid was extracted by magnetic beads-based method using automatic nucleic acid extraction system (MagPurix, Taiwan). Extracted nucleic acid was subjected to CT and NG detection using commercially available multiplex STD-real time PCR kit (Hybribio, Hong Kong). Real-time PCR was performed in reaction volume of 20ul composing of 17.5ul of PCR master mix, 0.5ul of Taq polymerase (5IU/ul) and 2ul of extracted DNA templates using Applied Biosystem 7900HT Fast Real-Time PCR system. This Real-time PCR was designed not only against the cryptic plasmid region of the CT but also could detect the new variant *Chlamydia trachomatis* (nvCT) 377bp deletion region, which enabled the RT-PCR kit to detect the majority kinds of CT strains. Reaction mixture was incubated at 95°C for 10 minutes before went through 45 amplification cycles of 95°C for 15 seconds and 60°C for 60 seconds. Positive florescence signal indicates the presence of the corresponding STI microorganisms. An optional on-site rapid screening test for syphilis was offered to all participants, with SD BIOLINE Syphilis 3.0 (multi), a one-step Anti-TP test kit.

### Statistical analysis

The prevalence was calculated among all sexually experienced and sexually active participants with 95% confidence intervals (CI). The “sexually experienced” were defined as those reported ever having sex, whilst the “sexually active” were those reported having one or more sex partners in the past 12 months. The sample was divided and analysed according to three age groups: young (18–26 years), middle (27–39 years), and older (40–49 years). Weighting was adjusted according to 2011 census data considering the variables of age, gender, and birthplace. Overall prevalence was computed by dividing the positive individual or committing cases by the total number of valid samples, unless specified, weighted data was used in the subsequent analyses.

Univariate analysis was first performed to assess the factors associated with CT infection. Those odds ratios (OR) with p ios (would then be included in the multivariable logistic regression adjusted for confounders including age and gender. The models were adjusted by comparing the Akaike information criterion (AIC) value out of the different combinations. Those variables with p<0.05 were considered statistically significant. Given the high *Chlamydia trachomatis* prevalence in female participants, a separate regression was performed when age was controlled. Different adjusted odds ratios (aORs) were estimated for both “sexually experienced” and “sexually active”. Analyses were conducted according to complex sampling design, using the svy module with Stata 14.1 (Stata Corp, College Station, Tex). To examine the homogeneity of CT prevalence in regions, which were categorised into Hong Kong Island and Kowloon Peninsula; and the first, second and third generation of new town in the territory, multilevel analyses were performed [33].

## Results

### Participants

Of the 3647 eligible addresses, 893 participants were recruited, of which 12 urine samples failed the control tests, these were therefore excluded from the final analyses. The overall response rate was 24.5%. Out of the remaining 881 urine samples (346 males and 535 females), 16 were positive for CT. The demographics and behavioural information of the participants are presented in S1 Table. On the whole, there was fairly even distribution of participants among the three age groups but female participants (52.8%) were slightly more represented than the males. About 75% of them attended senior high school or above and a quarter were born in mainland China, Macao or Taiwan. Women accounted for the majority of non-permanent residents as a result of Hong Kong’s migration policy that allows 150 people per day to enter Hong Kong for family union. About half of the participants in our sample were married or cohabitating and 60% of them had only one sex partner in the previous 12 months; another 4% had two or more sex partners in the same period. Among those who had “no sex partners” in the past 12 month, there were 57 married people reported no sexual intercourse with their spouse and hence grouped under the sexually experienced group.

### CT prevalence

The overall prevalence of CT among all 18–49 years old Hong Kong residents irrespective of their sexual practice was low at 1.4% (95% CI 0.8–2.5%) with 1.2% (95%CI 0.5–2.8%) for men and 1.7% (95%CI 0.9–3.1%) for women respectively. (S2 Table) However, prevalence was higher in the sexually experienced and the highest amongst the sexually active. The highest CT prevalence was found in the 18-26-year-old sexually active women (5.8%; 95% CI 1.7–18.2%), followed by 4.8% (95% CI 1.2–17.6%) in the sexually active men. The third highest group, however, was the 40-49-year-old sexually active women at 4.1% (95% CI 1.8–9.0%), giving rise to a U-shape distribution curve. Female participants in general had a higher prevalence of *Chlamydia trachomatis* than their male counterparts but no positive case of CT was found amongst the 30–39 years old women.

The spatial distribution of the positive cases and prevalence among the sexually active participants is shown in S1 Fig (drawn by one collaborator in ArcGIS 10.3.1 software). A core-peripheral distribution of *Chlamydia trachomatis* prevalence was observed in the territory. Those more established areas in Central & Western districts on Hong Kong Island, Tsuen Wan, Islands and Eastern were most prevalent with CT infection (>5%), surrounded by Sha Tin, Kwai Tsing and Sham Shui Po in Kowloon and New Territory West with estimated prevalence of *Chlamydia trachomatis* infection (3.1–5.0%) but much lower or no prevalence in the peripheral districts.

### Risk factors

The adjusted and crude ORs of CT infection are illustrated in S3 and S4 Tables. Among the sexually active, positive CT cases were independently associated with younger age (18–26 years old; aOR = 9.96; 95% CI 2.09–47.5); living alone (aOR = 11.90; 95% CI 1.89–75.1); and partners who had travelled out of Hong Kong in the previous 12 months (aOR = 5.35; 95% CI 1.25–22.8). For those sexually experienced women whose partners had travelled outside Hong Kong in the previous 12 months, they were about eight times (aOR = 7.73; 95% CI 1.66–35.9) more likely to be infected with chlamydia. The other two risk factors for CT infection among sexually active females were younger age (18–26) and living alone, with aOR at 25.4 (95% CI 2.81–230) and 8.99 (95% CI 1.46–55.4) respectively. A strong association was identified among women between CT infection and preference of public facilities if suspected of STIs with aOR at 7.65 (95% CI 1.96–29.9) among the sexually active female participants.

## Discussion

TeSSHS identifies CT prevalence among the general population in Hong Kong and maps it according to its geographical distribution. Contrast to the clinical findings from SHC, TeSSHS finds an overall low *Chlamydia trachomatis* prevalence in Hong Kong but has identified high prevalence in certain pockets of the population. A unique U-shape distribution of *Chlamydia trachomatis* infection in younger and older women was identified, which is very different from other population-based studies conducted elsewhere. It shows a hidden epidemic among the sexually active residents and finds travel history has significant roles to play in its transmission.

One main limitation is that TeSSHS has a relatively low response rate which is understandable given the sensitive nature of the study and is comparable to those reported in Natsal-3 [8]. One may also argue that perhaps those with higher perceived risks would be more forthcoming in participation. However, three indirect evidences may suggest otherwise. First, compared to the Chinese Health and Family Life Survey in 1999–2000, TeSSHS has a lower proportion of participants with ≥1 sex partners in the past 12 months i.e. 5.2%, (95% CI 3.3–8.2%) for males and 2.7% (95% CI 1.6–4.7%) for females compared to that of China study, 10.0% for males and 4.0% for females admitted so [16]. Secondly, compared to the Youth Sexuality Study (2011) in Hong Kong where 19.7% of male and 5.2% of female youth (18–27 years old) reported eposex partners in the past six months [34], the proportions of male and female aged 18–26 years old with ≥wisex partners in the past 12 months were 2.6% (95% CI 0.9–7.2%) and 3.3% (95% CI 1.1–9.7%) in TeSSHS respectively. Thirdly, the consistent condom use reported in the general population is 24% (95% CI 21.84–26.16%) [35] whilst the consistent condom use among TESSHS participants is 35.9%, indicating that they may be more health conscious and prepared to practise sexual risk reduction.

Another limitation was that no typing of PCR reactive samples was made in this project. Since most prevalence studies about chlamydial infections were carried out in Europe or USA, the ratio of distinct geno/sero types of *Chlamydia trachomatis* would be of high interest. Though the RT-PCR kit used could detect the great majority of CT strains, it could have missed some *Chlamydia trachomatis* strains that do not have any cryptic plasmid e.g. the new Swedish variant.

The overall prevalence of CT in Hong Kong i.e. 2.0% among female and 1.5% among male sexually experienced participants, is lower compared to those of China and global estimation but similar to those reported in European countries. In World Health Organization (WHO) global estimation of CT in 2012, the prevalence among 15–49 year old women is 4.2% (3.7–4.7%) [36] while in the Chinese Health and Family Life Survey, the prevalence equivalent is 2.6% (95% CI 1.6–4.1%) for females, and 2.1% (95% CI 1.3–3.3%) for males [16]. Findings in TESSHS is similar to the national prevalence study in France, e.g. CT prevalence is 1.6% (95% CI 1.0–2.5%) for women and 1.4% (95% CI 0.8–2.5%) for men between 18 and 44 years old in 2010 [11]. It is argued that the socioeconomic status and lifestyle of Hong Kong would be more comparable to that of developed countries [36].

This study also identifies that CT is more common among young individuals in Hong Kong, which is consistent with data from the USA [14], France [11], and the UK [8]. Among the sexually experienced, CT prevalence identified by TeSSHS is more comparable to the estimated prevalence among those aged 26 or below in the European countries with the pooled average prevalence at 3.6% (2.4–4.8%) for females and 3.5% (95% CI 1.9–5.2%) for males [37]. Other parts of the world, young females are also found with much higher estimated CT prevalence, e.g. in Peru, the CT prevalence in the 18–27 year-old sexually experienced women is estimated as high as 7.8% (95%CI 7.1–8.5%) [38].

At the same time, TeSSHS finds a higher prevalence of *Chlamydia trachomatis* with estimated prevalence of 4.1% (95% CI 1.8–9.0%) in the sexually active 40–49 year-old. All of them reported only one sex partner in the previous 12 months. These older women were mostly married and whose partners reportedly travelled out of Hong Kong within the past 12 months. TeSSHS also identifies partners travelling out of Hong Kong as a risk factor among the sexually experienced females, which is similar to that of the China CT study [16]. According to the data from SHC, the pattern of clinically CT cases shows two similar peaks for males, one at age 20–29 years and the other at age ≥40 years, whereas for females only the 20–29 years old peak is observed [39]. The mismatch of the SHC data and TeSSHS could be that the older females are normally reluctant to come forth for *Chlamydia trachomatis* testing as they may not be aware of their risks [4].

Naturally CT prevalence is much lower in the general populations than those in key populations. According to a study with cross-border Hong Kong-based Chinese male truck drivers in Hong Kong, CT prevalence is as high as 8.5% [40]. Another study among female sex workers in Hong Kong finds CT prevalence at 4.6% using CT DNA testing [41], which is higher than the estimation in the sexually experience but lower than that of the sexually active young women in our study. Nevertheless, a more recent CT prevalence estimation among the female sex workers in Hong Kong estimates the genital CT prevalence at 10.6% [28]. Over the Hong Kong-China border in Shenzhen, the combined prevalence of genital CT is reported to be as high as 17.7% in a sample of 2534 patients from 34 hospitals [42].

Other developed countries such as in the USA and UK have introduced opportunistic CT screening programs where young females are recommended to have CT tested regularly [8, 19]. A systematic review concludes that when prevalence of CT is between 3.1–10.0%, it can be cost-effective [43]. Based on the findings of TeSSHS, *Chlamydia trachomatis* screening for sexually active youth between 18–26 years of age and older women (40–49 years) could be cost-effective. Other risks factors such as living status and travel history could help identify people of higher risks. Certain key populations have expressed concern and the need of CT screening, for example, in the CT prevalence study among the cross-border truck drivers, a third of those who phoned for the results expressed this need [40]. The female CT positive participants indicate testing preference of public facilities, which could be related to costing issue, or their trust in public health system in Hong Kong. The public facilities could be ideal sites for health promotion, education and screening for chlamydia.

## Supporting information

S1 Fig The spatial distribution of the positive cases of *Chlamydia trachomatis* and its prevalence among the sexually active participants. (TIF)

S1 Table Unweighted observations and weighted distribution of the TeSSHS participants (N = 881). (DOCX)

S2 Table CT Prevalence estimated among all, sexually active and sexually experienced participants (N = 881). (DOCX)

S3 Table Factors associated with *Chlamydia trachomatis* infection by univariate and multivariable analyses. (DOCX)

S4 Table Factors associated with *Chlamydia trachomatis* infection among all the female participants. (DOCX)
