# Correction: Prevalence and risk factors of chlamydia infection in Hong Kong: A population-based geospatial household survey and testing

**DOI:** 10.1371/journal.pone.0199767

**Published:** 2018-07-17

**Authors:** William Chi Wai Wong, Yanping Zhao, Ngai Sze Wong, William L. Parish, Heidi Yin Hai Miu, Li-Gang Yang, Michael Emch, King Man Ho, Francois Yeung Fong, Joseph D. Tucker

The Data Availability statement for this article is incorrect. The correct statement is: Data cannot be publicly deposited due to the sensitive nature of the study and concerns over the participants’ privacy. The regulation on data confidentiality was from Health and Medical Research Fund (funding agency of this research). Interested researchers can request the data from Prof Cindy Lo Kuen Lam: Clinical Professor, Head of Department of Family Medicine and Primary Care, Department of Family Medicine and Primary Care, The University of Hong Kong. E-mail: clklam@hku.hk Tel: (852) 2518 5650.

## References

[1] Wong WCW, Zhao Y, Wong NS, Parish WL, Miu HYH, Yang L-G, et al (2017) Prevalence and risk factors of chlamydia infection in Hong Kong: A population-based geospatial household survey and testing. PLoS ONE 12(2): e0172561 10.1371/journal.pone.0172561 28225805 PMC5321413

