# Supplementary figures and images for: Prevalence and risk factors of chlamydia infection in Hong Kong: A population-based geospatial household survey and testing
Source: PLoS One. 2017 Feb 22;12(2):e0172561. doi: 10.1371/journal.pone.0172561 (PMC5321413; doi:10.1371/journal.pone.0172561)

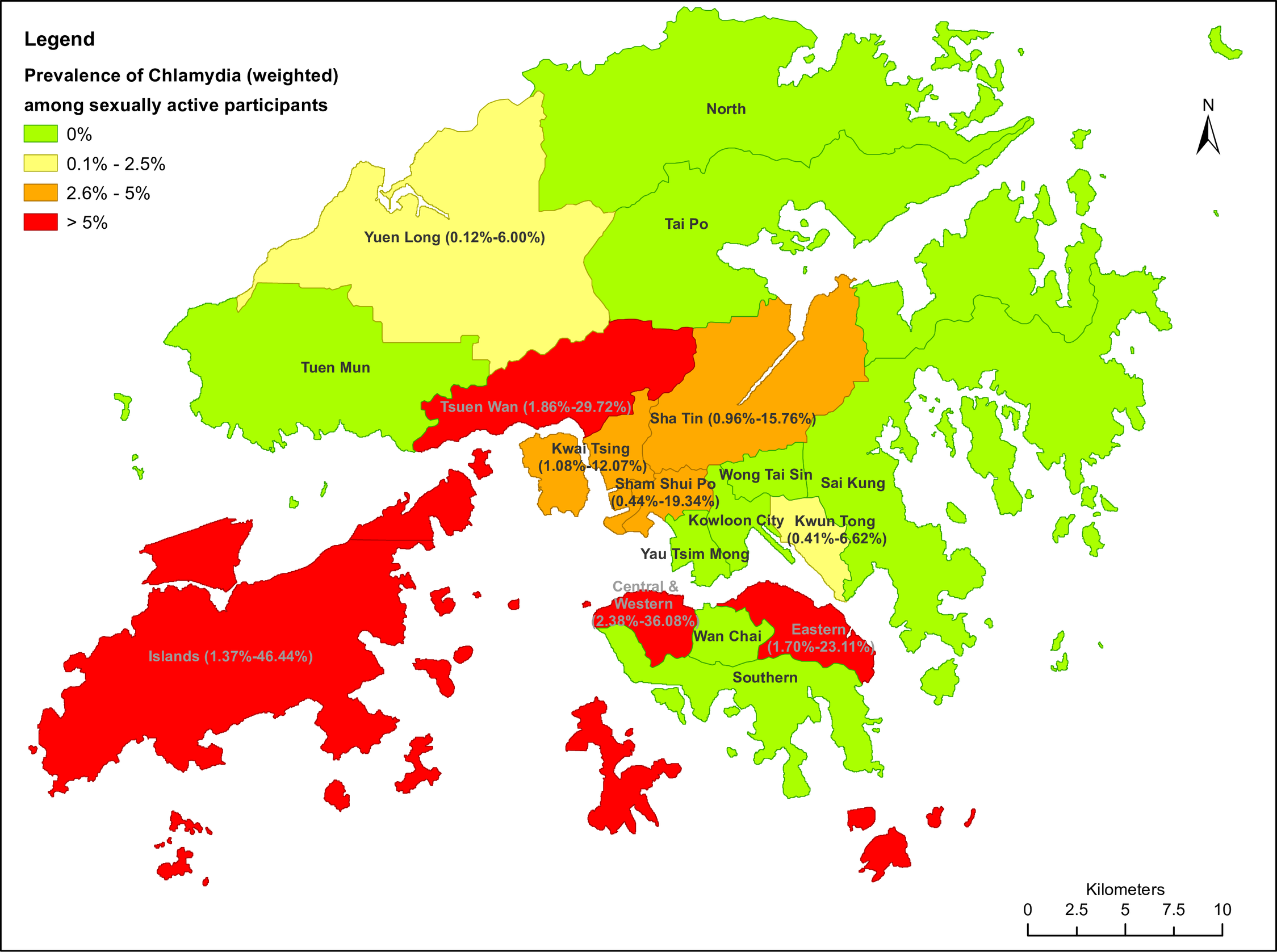

Supplement: S1 Fig — (TIF) [file pone.0172561.s001.tif]
