# Supplementary material for: Prevalence and risk factors of chlamydia infection in Hong Kong: A population-based geospatial household survey and testing
Source: PLoS One. 2017 Feb 22;12(2):e0172561. doi: 10.1371/journal.pone.0172561 (PMC5321413; doi:10.1371/journal.pone.0172561)
Supplement: S1 Table — (DOCX) [file pone.0172561.s002.docx]

| **Table 1. Unweighted observations and weighted distribution of the TeSSHS participants (N=881)** | | | | | | |
| --- | --- | --- | --- | --- | --- | --- |
|  | **Unweighted observations, No.** | | | **Weighted distributions, %** | | |
|  | Male | Female | All | Male | Female | All |
| **Characteristics** | 346 | 535 | 881 | 47.2 | 52.8 | 100 |
| **Age, y** | |  |  |  |  |  |
| 18 to 29 | 146 | 152 | 298 | 35.4 | 31.4 | 33.3 |
| 30 to 39 | 91 | 158 | 249 | 29.6 | 31.3 | 30.6 |
| 40 to 49 | 109 | 225 | 334 | 35.0 | 37.3 | 36.1 |
| **Education** | |  |  |  |  |  |
| <=Junior high school | 69 | 181 | 250 | 20.7 | 28.0 | 24.5 |
| =Senior high school | 115 | 183 | 298 | 34.0 | 36.0 | 35.1 |
| >Senior high school | 162 | 171 | 333 | 45.3 | 36.0 | 40.4 |
| **Birthplace** | |  |  |  |  |  |
| Hong Kong | 249 | 269 | 518 | 77.1 | 67.8 | 72.2 |
| Mainland/Macao/Taiwan | 93 | 263 | 356 | 21.7 | 31.4 | 26.8 |
| Other | 4 | 3 | 7 | 1.2 | 0.8 | 1.0 |
| **Residency^** | |  |  |  |  |  |
| Permanent ID | 335 | 472 | 807 | 97.3 | 91.6 | 94.3 |
| Non-Permanent ID | 11 | 63 | 74 | 2.7 | 8.4 | 5.7 |
| **Working Status** | |  |  |  |  |  |
| Working | 269 | 342 | 611 | 79.9 | 64.9 | 72.0 |
| Not working | 77 | 193 | 270 | 20.1 | 35.1 | 28.0 |
| **Marital Status** | |  |  |  |  |  |
| Single | 204 | 179 | 383 | 56.1 | 39.5 | 47.4 |
| Married/ cohabiting | 134 | 309 | 443 | 41.4 | 52.3 | 47.2 |
| Widowed/divorced/separated | 8 | 47 | 55 | 2.5 | 8.1 | 5.5 |
| **No. of sex partners in the past 12 months** | |  |  |  |  |  |
| 0~ | 133 | 184 | 317 | 37.1 | 36.7 | 36.9 |
| 1 | 195 | 338 | 533 | 57.7 | 60.6 | 59.2 |
| >=2 | 18 | 13 | 31 | 5.2 | 2.7 | 3.9 |
| **Number of people living with** | |  |  |  |  |  |
| 0 | 20 | 15 | 35 | 6.4 | 3.4 | 4.8 |
| 1-2 | 131 | 182 | 313 | 37.5 | 33.3 | 35.3 |
| More than 2 | 195 | 338 | 533 | 56.1 | 63.3 | 59.9 |
| **Partner travelled out of HK in the past 12 months*** | |  |  |  |  |  |
| Yes | NA | 211 | NA | NA | 36.9 | NA |
| No | NA | 324 | NA | NA | 63.1 | NA |
| * Only to be answered by female participants (please note that “No” here includes “partner didn’t leave” and “no partner” and “sexually inactive”. | | | | | | |
| ^ Permanent resident is defined in article 24 of the Hong Kong Basic Law and paragraph 2 of schedule 1 to the Immigration Ordinance, it includes those born in HK and those live in Hong Kong for more than seven years. | | | | | | |
