# Supplementary material for: Prevalence and risk factors of chlamydia infection in Hong Kong: A population-based geospatial household survey and testing
Source: PLoS One. 2017 Feb 22;12(2):e0172561. doi: 10.1371/journal.pone.0172561 (PMC5321413; doi:10.1371/journal.pone.0172561)
Supplement: S2 Table — (DOCX) [file pone.0172561.s003.docx]

**Table 2. CT Prevalence estimated among all, sexually active and sexually experienced participants (N=881)**

|  | All (N=881) (95%CI) | Sexually experienced^1^ (n=733) 95%CI | Sexually active^2^ (n=566) (95%CI) |  |
| --- | --- | --- | --- | --- |
| Prevalence of CT (all) | 1.4% (0.8-2.5) | 1.8% (1.0-3.0) | 2.3% (1.3-3.9) |  |
| 18-26 | 1.9% (0.8-4.7) | 3.4% (1.4-8.0) | 5.3% (2.1 -12.6) |  |
| 27-39 | 0.8% (0.2-2.4) | 0.9% (0.3-2.8) | 1.0% (0.3-3.3) |  |
| 40-49 | 1.8% (0.8-4.0) | 1.9% (0.9-4.2) | 2.5% (1.1-5.6) |  |
| **Male (n=346)** | 1.2% (0.5-2.8) | 1.5% (0.6-3.6) | 1.9% (0.8-4.4) |  |
| 18-26 | 1.7% (0.4-6.7) | 3.3% (0.8-12.0) | 4.8% (1.2-17.6) |  |
| 27-39 | 1.3% (0.3-5.1) | 1.6% (0.4-6.2) | 1.8% (0.4-7.0) |  |
| 40-49 | 0.6% (0.1-4.6) | 0.7% (0.1-4.8) | 0.9% (0.1-6.1) |  |
| **Female (n=535)** | 1.7% (0.9-3.1) | 2.0% (1.0-3.7) | 2.6% (1.4-4.9) |  |
| 18-26 | 2.2% (0.7-6.9) | 3.5% (1.1-11.0) | 5.8% (1.7-18.2) |  |
| 27-39 | 0.3% (0-2.3) | 0.4% (0.1-2.6) | 0.4% (0.1-3.1) |  |
| 40-49 | 2.8% (1.2-6.2) | 2.9% (1.3-6.5) | 4.1% (1.8-9.0) |  |

1. Sexually experienced: reported ever having sex
2. Sexually active: reported having sex in the last 12 months
