# Supplementary material for: Prevalence and risk factors of chlamydia infection in Hong Kong: A population-based geospatial household survey and testing
Source: PLoS One. 2017 Feb 22;12(2):e0172561. doi: 10.1371/journal.pone.0172561 (PMC5321413; doi:10.1371/journal.pone.0172561)
Supplement: S3 Table — (DOCX) [file pone.0172561.s004.docx]

**Table 3. Factors associated with *Chlamydia trachomatis* infection by univariate and multivariable analyses**

|  |  | |  | |  | |  | |  | |  | |  | |  | |
| --- | --- | --- | --- | --- | --- | --- | --- | --- | --- | --- | --- | --- | --- | --- | --- | --- |
|  | Univariate | | | | Multivariable | | | | | | | | | | | |
|  | All | | | | All | | | | Sexually | | | | Sexually active | | | |
|  |  | |  | |  | |  | | experienced (ever) | | | | (in last 12 months) | | | |
| Factors | OR | | 95% CI | | aOR | | 95% CI | | aOR | | 95% CI | | aOR | | 95% CI | |
| Male travelled out of Hong Kong (no) | 1.00 | |  | | 1.00 | |  | | 1.00 | |  | | 1.00 | |  | |
| Yes | 8.15 | | (2.25-29.54)** | | 11.1 | | (2.65-46.6)** | | 8.40 | | (2.10-33.5)** | | 5.35 | | (1.25-22.8)* | |
| Born in HK? (yes) | 1.00 | |  | | 1.00 | |  | | 1.00 | |  | | 1.00 | |  | |
| No | 4.44 | | (1.39-14.18)* | | 3.79 | | (0.96-15.0)+ | | 3.32 | | (0.89-12.4)+ | | 3.21 | | (0.82-12.6)+ | |
| Lives with (>2 others) | 1.00 | |  | | 1.00 | |  | | 1.00 | |  | | 1.00 | |  | |
| 0 (alone) | 6.21 | | (1.11-34.58)* | | 12.1 | | (1.85-79.1)** | | 12.3 | | (1.93-78.8)** | | 11.9 | | (1.89-75.1)** | |
| 1 or 2 other | 2.16 | | (0.67-7.01) | | 2.12 | | (0.74-6.09) | | 2.15 | | (0.74-6.24) | | 2.21 | | (0.71-6.90) | |
| STI testing facilities (private) | 1.00 | |  | | 1.00 | |  | | 1.00 | |  | | 1.00 | |  | |
| public | 2.69 | | (0.93-7.79) | | 2.50 | | (0.78-8.04) | | 2.67 | | (0.84-8.46)+ | | 2.77 | | (0.97-7.91)+ | |
| Age (27-39 years) | 1.00 | |  | | 1.00 | |  | | 1.00 | |  | | 1.00 | |  | |
| 18-26 years | 2.55 | | (0.57-11.28) | | 6.82 | | (1.36-34.2)* | | 8.04 | | (1.67-38.6)** | | 9.96 | | (2.09-47.5)** | |
| 40-49 years | 2.38 | | (0.58-9.71) | | 3.32 | | (0.72-15.2) | | 3.24 | | (0.72-14.7) | | 3.67 | | (0.80-16.8)+ | |
| Male | 1.00 | |  | | 1.00 | |  | | 1.00 | |  | | 1.00 | |  | |
| female | 1.43 | | (0.47-4.31) | | 2.27 | | (0.74-6.96) | | 1.87 | | (0.65-5.35) | | 1.78 | | (0.62-5.16) | |
| Observations (unweighted) | 881 |  | | 881 | |  | | 733 | |  | | 566 | |  | |  |
|  |  | |  | |  | |  | |  | |  | |  | |  | |
