# Supplementary material for: Prevalence and risk factors of chlamydia infection in Hong Kong: A population-based geospatial household survey and testing
Source: PLoS One. 2017 Feb 22;12(2):e0172561. doi: 10.1371/journal.pone.0172561 (PMC5321413; doi:10.1371/journal.pone.0172561)
Supplement: S4 Table — (DOCX) [file pone.0172561.s005.docx]

**Table 4. Factors associated with *Chlamydia trachomatis* infection among all the female participants**

|  | | |  |  | | | | |  |  |  |  |  |  |  |  |
| --- | --- | --- | --- | --- | --- | --- | --- | --- | --- | --- | --- | --- | --- | --- | --- | --- |
|  | | |  | Univariate ORs | |  | Multivariable aORs | | | | | | | | | |
|  | | |  | All | |  | All | | | | | | Sexually | | Sexually active | |
|  | | |  |  |  |  |  |  | | | | | experienced (ever) | | (in last 12 months) | |
| Factors | | |  | OR | 95% CI |  | aOR | 95% CI | | | | | aOR | 95% CI | aOR | 95% CI |
| Male travelled out of HK (no) | | |  | 1.00 |  |  | 1.00 |  | | | | | 1.00 |  | 1.00 |  |
| yes | | |  | 7.40 | (1.78-30.75)** |  | 10.3 | (2.06-51.2)** | | | | | 7.73 | (1.66-35.9)** | 4.84 | (0.92-25.6)+ |
| Born in HK? (yes) | | |  | 1.00 |  |  | 1.00 |  | | | | | 1.00 |  | 1.00 |  |
| no | | |  | 0.43 | (0.11-1.68) |  | 2.05 | (0.44-9.61) | | | | | 2.00 | (0.44-9.13) | 1.99 | (0.41-9.70) |
| Lives with (>2 others) | | |  | 1.00 |  |  | 1.00 |  | | | | | 1.00 |  | 1.00 |  |
| 0 (alone) | | |  | 6.66 | (0.62-71.34) |  | 10.8 | (1.79-65.6)* | | | | | 10.00 | (1.73-58.2)* | 8.99 | (1.46-55.4)* |
| 1 or 2 other | | |  | 1.60 | (0.41-6.21) |  | 1.51 | (0.49-4.64) | | | | | 1.44 | (0.47-4.40) | 1.44 | (0.42-4.93) |
| STI testing prefer (private) | | |  | 1.00 |  |  | 1.00 |  | | | | | 11.00 |  | 1.00 |  |
| public | | |  | 7.98 | (1.90-33.54)** |  | 8.15 | (1.88-35.4)** | | | | | 8.12 | (1.91-34.5)** | 7.65 | (1.96-29.9)** |
| Age (27-39) | | |  | 1.00 |  |  | 1.00 |  | | | | | 1.00 |  | 1.00 |  |
| 18-26 | | |  | 6.91 | (0.68-70.33) |  | 18.5 | (1.40-243)* | | | | | 20.9 | (1.83-240)* | 25.4 | (2.81-230)** |
| 40-49 | | |  | 8.83 | (0.98-79.93) |  | 11.4 | (1.06-123)* | | | | | 11.00 | (1.01-119)* | 11.9 | (1.10-128)* |
| Observations (all, unweighted) | | |  | 535 |  |  | 535 |  | | | | | 468 |  | 351 |  |
|  |  |  |  |  |  |  |  |  |  |  |  |  |  |  |  |  |
